# Engineering Dark Chromoprotein Reporters for Photoacoustic Microscopy and FRET Imaging

**DOI:** 10.1038/srep22129

**Published:** 2016-03-01

**Authors:** Yan Li, Alex Forbrich, Jiahui Wu, Peng Shao, Robert E. Campbell, Roger Zemp

**Affiliations:** 1 Department of Chemistry, University of Alberta, Edmonton, Alberta, Canada T6G 2G2; 2 Department of Electrical & Computer Engineering, University of Alberta, Edmonton, Alberta, Canada T6G 2V4

## Abstract

A subset of the family of fluorescent proteins are the non-fluorescent chromoproteins which are promising probe molecules for use in photoacoustic imaging and as acceptor chromophores in Förster resonance energy transfer (FRET)-based biosensors. Typical approaches for fluorescent protein optimization by screening of large libraries of variants cannot be effectively applied to chromoproteins due to their characteristic lack of fluorescence. To address this challenge, we have developed a directed evolution method to iteratively screen large libraries of protein variants on the basis of their photoacoustic signal levels. By applying this procedure to the promising Ultramarine and cjBlue chromoprotein templates, we were able to identify improved variants with a 02–04 fold increase in photoacoustic signal-to-noise ratio after only a few evolutionary steps. These improved variants enable more accurate spectral de-mixing and localization of protein-producing bacteria *in vivo* and serve as effective FRET acceptors for both fluorescence- and photoacoustic-based detection of protease activity.

Fluorescent proteins (FPs), and various genetically-encoded optical reporters based on FPs, have revolutionized optical imaging and our ability to visualize biological processes in cells and organisms^1^. Essentially all of the FPs in widespread use have undergone substantial amounts of protein engineering aimed at increasing their quantum yield, extinction coefficient, folding efficiency, photostability, and other key properties. However, there are some emerging applications where a high quantum yield is detrimental to the performance of the reporter protein, and non-fluorescent (or only weakly fluorescent) FP homologues, otherwise known as chromoproteins (CP), are preferred. Examples of such applications include photoacoustic (PA) imaging and Förster resonance energy transfer (FRET) with a dark acceptor.

PA imaging relies on photothermal conversion to produce acoustic signals detectable by ultrasound transducers. Accordingly, reporter chromophores with high optical absorption and low quantum yield typically provide the highest signal-to-noise ratio (SNR). This emerging technique has been applied for visualizing blood oxygenation^2^,^3^,^4^, angiogenesis^5^,^6^, gene expression^7^,^8^,^9^,^10^, and optical contrast agents^11^,^12^. One implementation of PA imaging is acoustic-resolution photoacoustic microscopy (AR-PAM), in which ultrasound transducers are used to detect acoustic signals generated due to laser pulses. Notably, AR-PAM is capable of imaging to depths of a few millimeters to centimeters in tissue with relatively high resolution. PA imaging represents an important intermediary between superficial optical microscopy and deep tissue imaging and is a promising strategy for reflection-mode *in vivo* molecular imaging.

While endogenous hemoglobin^2^,^5^ and melanin^6^ are two ubiquitous chromophores that enable non-invasive and label-free PA imaging, researchers are increasingly exploring the use of exogenous reporter chromophores. For example, as a principle enzyme in melanin production, tyrosinase has been used as a genetically-encoded reporter molecule^7^,^8^,^9^,^10^,^13^. However, high levels of unpackaged intracellular melanin precursors may be cytotoxic^7^. β-galactosidase, encoded by the *LacZ* gene, has also been used as a reporter molecule for PA imaging of gliosarcoma tumor cells^14^,^15^,^16^. Unfortunately, previous studies with β-galactosidase have relied on the substrate X-gal, which was not designed for *in vivo* use and can induce unwanted side-effects^7^. Recently, FPs have been demonstrated to have potential utility as PA reporter molecules^17^,^18^,^19^. Razansky *et al.*^17^ demonstrated that PA tomography could detect tissue-specific expression of enhanced green FP (EGFP) and mCherry FPs several millimeters deep in tissues while maintaining 20–100 μm resolution. In 2012, an infrared FP, engineered from a bacteriophytochrome that binds a biliverdin chromophore, was used for PA imaging *in vivo*^18^. In 2014, Krumholz *et al.*^19^ demonstrated PA imaging of an infrared FP at a tissue depth of 8 mm with sub-millimeter resolution. Even with these promising results, the characteristic ability of FPs to fluoresce necessarily diminishes their photothermal conversion efficiency and thereby reduces their utility for PA imaging.

Ultramarine^20^ and cjBlue^21^ are CPs that absorb orange-red light with high extinction coefficients (>60,000 M^−1^cm^−1^) and low fluorescence quantum yields (<0.001), making them promising candidates for PA imaging. Laufer *et al.*^22^ quantified the PA signals from various FPs including cjBlue and found that, relative to FPs, CPs gave greater PA signal and were more robust to laser-induced bleaching after repetitive laser exposures.

Just as engineered FPs have led to major advances in live cell fluorescence imaging, engineering and optimization of CPs for PA imaging could lead to improved SNR for *in vivo* imaging and help to realize the full potential of PA imaging. We further envisioned that optimized CPs would also be of great utility as FRET acceptors for both fluorescence and PA imaging. FRET is a distance dependent phenomenon in which the fluorescence of a higher energy donor fluorophore is quenched due to energy transfer to a lower energy acceptor chromophore, at distances of less than ~10 nm. One limitation of FP-based FRET measurements is that it is challenging to perform multiplexed measurements using two different FRET pairs, due to the broad excitation and emission spectra typically associated with FPs^23^. To overcome this limitation, non-fluorescent CPs have been utilized as dark acceptors. FRET to a non-fluorescent, or ‘dark’, acceptor could also be used in conjunction with PA imaging since the energy transferred to the dark acceptor may lead to thermoelastic expansion and PA emission due to the generation of heat. PA imaging of FRET could provide deep-tissue, high-resolution visualization of protein-protein interactions. Several examples of PA-based FRET-imaging have been reported^24^,^25^.

As a step towards the development of optimized CPs for PA imaging, we developed a method to screen large libraries of CPs for enhanced PA characteristics. This method is analogous to the fluorescence screening and directed evolution techniques that are commonly used in FP engineering (Fig. 1a). Specifically, the Ultramarine and cjBlue templates were subjected to multiple rounds of directed evolution to produce several fold increases in PA signal. Using the improved variants, we demonstrate multiwavelength PA imaging in mammalian tissue and application as FRET acceptors for both fluorescence and PA imaging.

## Results

### Photoacoustic and optical properties of FPs and CPs

We used mCherry^26^,^27^, EYFP^28^, and a dark YFP-based CP (REACh)^29^ as representative FPs to compare to the chromoproteins cjBlue^21^ and Ultramarine^20^ in terms of photostability, SNR, extinction coefficient, quantum yield, and absorption spectrum (Supplementary Table 1). Similar to Laufer *et al.*^22^, we found that cjBlue and Ultramarine gave the highest PA signals, which is attributed to the lower quantum yield and hence more efficient photothermal conversion. Furthermore, cjBlue and Ultramarine were less susceptible to photobleaching compared to the other three proteins (Supplementary Fig. 1). The SNR decreased by 25–50% for the two FPs and REACh and less than 10% for cjBlue and Ultramarine, following 1000 laser pulses at 2.5 mJ/cm^2^. Overall, the enhanced photostability and the higher PA signals generated by cjBlue and Ultramarine suggested that these CPs are promising tools for PA imaging.

Based on the measured fluence, the concentration, and the SNR (Supplementary Table 1), we estimate that imaging at the ANSI (American National Standards Institute) limit of ~20 mJ/cm^2^ should enable imaging of the Ultramarine and cjBlue CPs at concentrations of 200 nM and 600 nM, respectively, with ~10 B SNR. These concentrations agree to within an order of magnitude with Li *et al.*^14^ who demonstrated 515 nM detection sensitivity of the blue product (5,5′-dibromo−4,4′-dichloro-indigo) from the LacZ gene and Filonov *et al.*^18^ who demonstrated 16 μM detection sensitivity of an infrared FP (iRFP) sample embedded in tissue. It should be noted that LacZ provides enzymatic amplification leading to significant accumulation of a blue product, while FPs and CPs have no amplification mechanism.

Multiwavelength PA imaging of Ultramarine and cjBlue revealed that the PA spectrum closely resembled the absorption spectrum (Supplementary Fig. 2) and that spectral demixing techniques could accurately separate the CPs from blood (Supplementary Fig. 3). We observed only slight cross-talk between the Ultramarine and blood signals, most likely due to the similar absorption spectra of Ultramarine and deoxyhemoglobin at wavelengths greater than 585 nm. Two-dimensional multi-modal ultrasound-photoacoustic B-scans across a site where *E. coli*, expressing either Ultramarine (Supplementary Fig. 4a,b) or cjBlue (Supplementary Fig. 4c,d), had been injected into the hind flank of a rat, demonstrated that spectral unmixing could enable these CPs to be detected at depths of greater than 1 mm in tissue.

Given the characteristics of the transducer and the signal detected from the phantom imaging (Supplementary Fig. 3), we estimate that the minimum concentration of cells required to give ~10 dB SNR at the ANSI limit is 100–1,000 cells per voxel. This is consistent with the signal from the *in situ* imaging experiment (Supplementary Fig. 4) which had a SNR of 25 dB an injection of ~2,000 cells/voxel (~10^9^cells/mL injection) which was ~1 mm below the tissue surface. This is similar to Razansky *et al.*^17^ who demonstrated that the minimum number of cells is ~10^3^ for imaging DsRed-expressing HeLa cells.

### Directed evolution of CPs for improved photoacoustic characteristics

**Figure 1:**
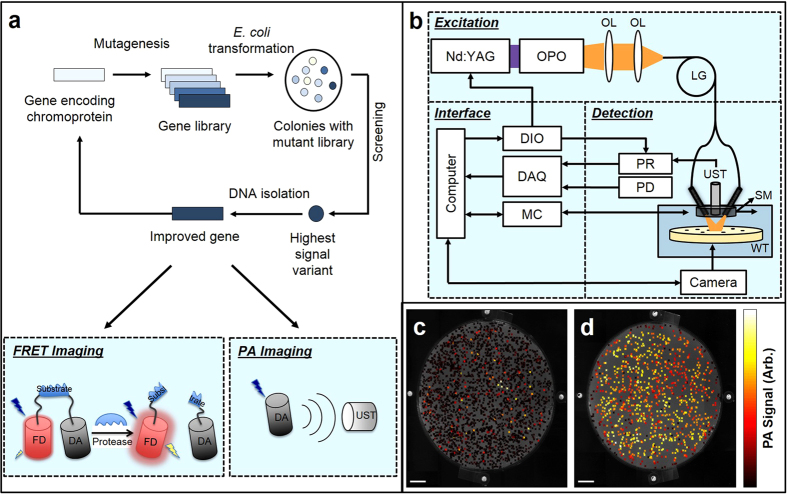
Directed evolution with PA-based screening. (**a**) Directed evolution protocol. Random mutations are introduced into the CP gene to form a gene library, which is then used to transform *E. coli* cells, which are grown as colonies on agar plates. The colonies are screened either visually or for PA signal generation, and the best colonies are used as templates for the subsequent round of library generation. (**b**) PA system schematic. OPO, optical parametric oscillator; OL, objective lens; LG, light guide; UST, ultrasound transducer; PD, photodiode; PR, pulser-receiver; DIO, digital input-output card; DAQ, data acquisition card; MC, motor controller; SM, motor stage; WT, water tank. (**c,d)** Directed evolution of Ultramarine. PA imaging of a typical plate of *E. coli* colonies expressing the library resulting from error-prone PCR of the Ultramarine DNA (**c**), or enhanced variants after several iterative rounds of screening (**d**). Grayscale background image represents the camera image of the agar plate, while the red pseudocolor represents the PA overlay. Scale bar represents 1 cm.

As our initial data demonstrated that CPs are promising genetically-encoded chromophores for PA imaging, we were motivated to develop a screening protocol to enable the directed evolution of CPs for improved PA signal generation. Briefly, libraries of gene variants were produced by error-prone PCR and used to transform *E. coli* cells that were then grown as colonies on agar plates under conditions that induced protein expression. These libraries were screened by a primary direct visual inspection and a secondary *in vitro* absorbance measurement (absorbance-based screening), or by PA -based screening. For the latter strategy, single colonies were located using a camera, a single element transducer was automatically positioned overtop the colony, and laser-induced PA signals were detected (Fig. 1b). The lateral resolution for PA imaging (~125 μm) was finer than the size of the typical colony (0.7–1 mm). As represented in Fig. 1c, a typical gene library produced by error-prone PCR includes many colonies that exhibit a loss-of-function with respect to absorbance (represented as the black-colored colonies). However, a select few colonies have high signal levels (represented as yellow to white-colored colonies). Colonies with high SNR were picked and used as the template for the subsequent round of library creation and screening. After three iterations, all high-signal producing colonies were plated on a single agar dish for imaging (Fig. 1d). To test the sensitivity of PA-based screening, the variation of the PA signal from uniformly transfected plates of *E. coli* colonies was quantified as 10–13%. This variation may be due to variations in the colony size, stability of the protein, or photobleaching.

**Figure 2:**
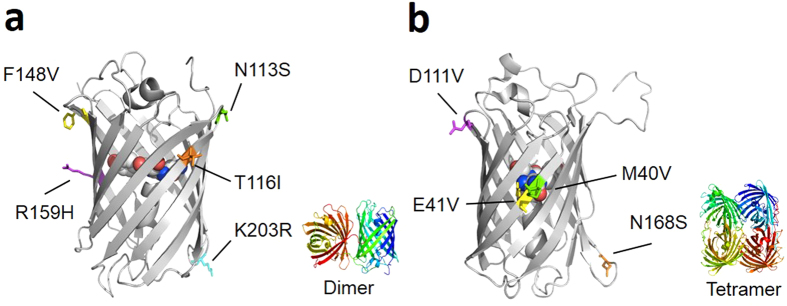
Location of substitutions in dUltramarine2 and cjBlue2 that were introduced during the directed evolution process. The X-ray crystal structure of Rtms5 (PDB ID 1MOU)^30^ is used here to represent Ultramarine (**a**). The monomeric cjBlue subunit is shown in (**b**) (PDB ID 2IB5)^21^.

For Ultramarine, four iterations of absorbance-based screening followed by three iterations of PA-based screening were performed. Similarly, three iterations of absorbance-based screening followed by four iterations of PA-based screening were performed for cjBlue. These efforts led to the identification of improved dUltramarine2 (Fig. 2a) and cjBlue2 (Fig. 2b) variants, with 5 (N113S, T116I, F148V, R159H, and K203R) and 4 (M40V, E41V, D111V, and N168S) mutations, respectively (Supplementary Fig. 5). Colonies of *E. coli* expressing these variants were vibrantly colored when viewed under normal illumination conditions just 12 hours after transformation (Supplementary Fig. 6). Based on its structural homology with Rtms5^30^, all five mutations of the Ultramarine variant were found to occur at residues with side chains directed towards the exterior of the protein. As the R159H mutation occurs in the former dimerization interface of Ultramarine^29^, we suspected that the originally monomeric Ultramarine may have reverted to a dimeric form. Indeed, this suspicion was confirmed by gel filtration chromatography (Supplementary Fig. 7). Of the 4 mutations in cjBlue (Fig. 2b), one is internal to the β-barrel (E41V) and three are surface mutations (M40V/D111V/N168S). Gel filtration chromatography confirmed that these mutations did not change the native tetrameric quaternary structure of the protein (Supplementary Fig. 7). The final variants were designated as dUltramarine2 (the d prefix indicates the dimeric structure) and cjBlue2. To effectively convert dUltramarine2 into a “monomer”, we constructed a tandem dimer (td) variant (Supplementary Fig. 8) as previously described^26^. The key photophysical characteristics of the variants are provided in Supplementary Table 2.

**Figure 3:**
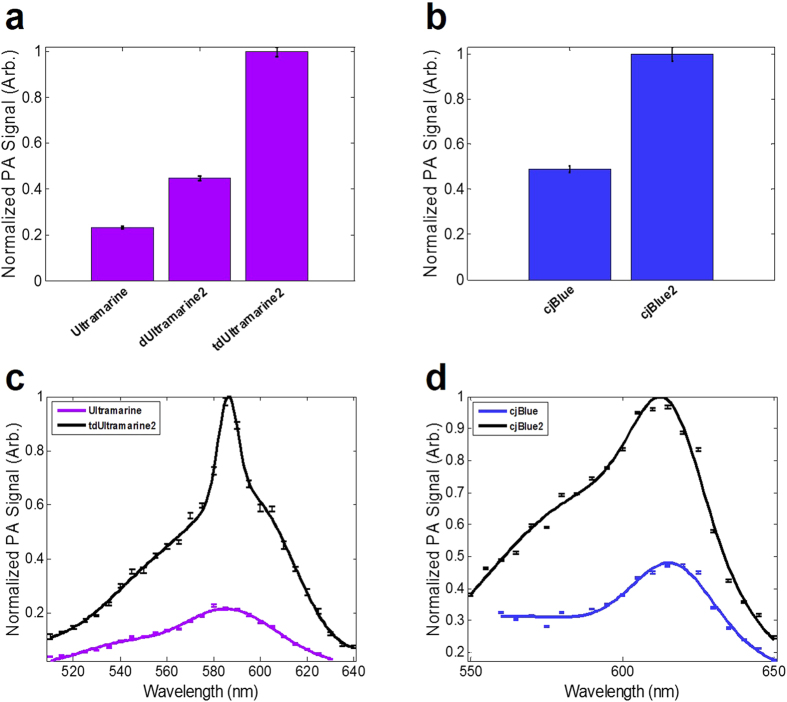
PA characteristics of enhanced CP variants. (**a**,**b**) represent the SNR of solutions of purified Ultramarine and cjBlue and variants, respectively. PA signal was calibrated to account for slight variations in pulse-to-pulse fluence (2.5 ± 0.4 mJ/cm^2^) and protein concentration (~100 μM). (**c**,**d**) represent the PA spectra of tdUltramarine2 and cjBlue2 variants compared to the original, respectively. The mean is determined from three trials with each trail using at least 20 laser pulses at each wavelength. Error bars represent the standard error of the mean (SEM).

PA measurements on solutions of purified Ultramarine, dUltramarine2, tdUltramarine2, cjBlue, and cjBlue2 revealed substantial improvements in the SNR on a per molecule basis (Fig. 3). Relative to Ultramarine, dUltramarine2 has a 1.9-fold increase in PA SNR, a 27% increase extinction coefficient, and at least a 2-fold lower quantum yield. On a per molecule basis (i.e., 2 chromophores per tandem dimer), tdUltramarine2 has a 4.3-fold greater PA signal, a more than 3-fold higher extinction coefficient, and a 2-fold lower quantum yield. Relative to cjBlue, the cjBlue2 variant has a greater than 2-fold increase in PA signal, and a slight blue shift, with most other photophysical characteristics remaining largely unchanged. The PA spectra of the improved variants closely resembled the original proteins (Fig. 3c,d).

**Figure 4:**
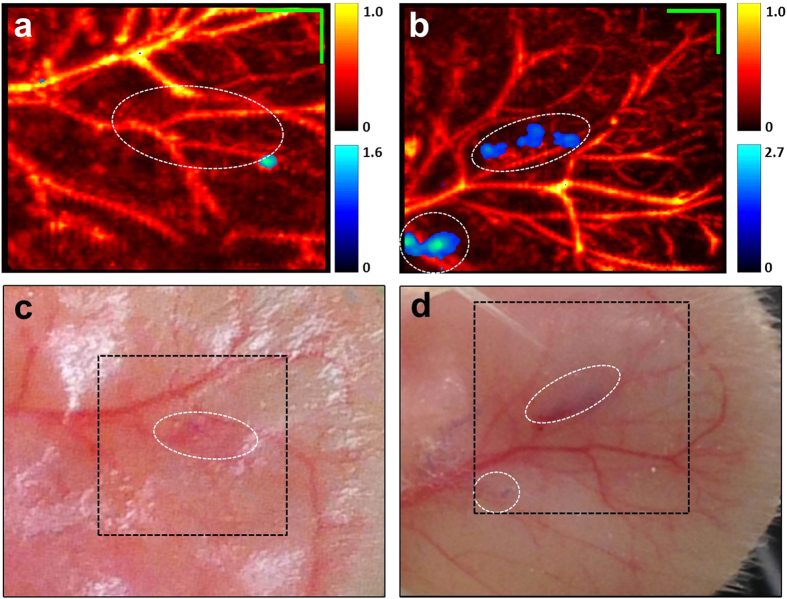
*In vivo* imaging of Ultramarine and tdUltramarine2. Ultramarine (**a**) and tdUltramarine2 (**b**) *E. coli* pellet injected directly into the ear of a rat and the corresponding photographic images (**c**,**d**), respectively. Green scale bar represents 1 mm. The red-yellow and the blue-green colorbars represent the unmixed and normalized photoacoustic signals due to hemoglobin and chromoprotein, respectively.

As tdUltramarine2 provides the largest PA signal on a per molecule basis, we further investigated the utility of this protein for *in vivo* imaging. Accordingly, we injected *E. coli* cells expressing Ultramarine or tdUltramarine2 into the ear of an anaesthetized rat. Each injection consisted of approximately 10 μL of 10^7^–10^8^ *E. coli* cells/ml. Multiwavelength imaging was conducted to compare SNR *in vivo* and assess the utility of spectral unmixing to distinguish the new CP variants from blood (Fig. 4). Although our phantom studies (Fig. 3) revealed that Ultramarine variants had 3–4 fold greater photoacoustic signal compared with Ultramarine at 585 nm, the peak spectrally unmixed tdUltramarine2 signal in injected regions *in vivo* was on average only 2.7 ± 1.1 fold greater than the spectrally unmixed Ultramarine (p < 0.05, N = 4).

SNR after spectral unmixing may not be as high as the peak pre-unmixed signal at 585 nm since the unmixing process involves data at other wavelengths where absorption is not as strong, and noise propagation through the unmixing process will depend on the condition number of the molar extinction coefficient matrix. Some variability was seen with the *in vivo* data, likely associated with different subdermal spread of injected cells, however, Fig. 4 illustrates significantly higher average signal from tdUltramarine2-expressing cells compared to wild-type Ultramarine-expressing cells.

### Characterization of new CPs as FRET acceptors

Although it was optimized for its PA signal and not necessarily for use as a FRET acceptor, we expected tdUltramarine2 could serve as a dark acceptor for a range of FRET donors due to its high extinction coefficient (203,400 M^−1^cm^−1^). To explore the use of tdUltramarine2 as a FRET acceptor, we genetically fused it to EGFP, mPapaya1^31^, and mRuby2^32^ via a 10-amino acid linker to create a set of 3 fusion proteins. Analogous constructs were made using Ultramarine in place of tdUltramarine2. The absorption spectra of the purified fused FRET pairs (Supplementary Fig. 9) were used to determine the stoichiometry of each pair. These calculations revealed that the stoichiometries were generally close to unity, as expected for genetically fused FRET pairs (Supplementary Table 3). SDS-PAGE analysis revealed no substantial amount of proteolysis of the purified proteins (Supplementary Fig. 10b). We attribute deviations from unity to differences in the maturation efficiency (i.e., the fraction of proteins that generate a chromophore). The primary outlier was the mRuby2-Ultramarine fusion with an apparent 4.6 copies of Ultramarine for every copy of mRuby2, indicating poor maturation efficiency for mRuby2 in the context of this particular fusion. Calculation of the Förster radii (R_o_) for all pairs revealed that tdUltramarine2 should be a substantially better FRET acceptor than Ultramarine (Supplementary Table 3).

**Figure 5:**
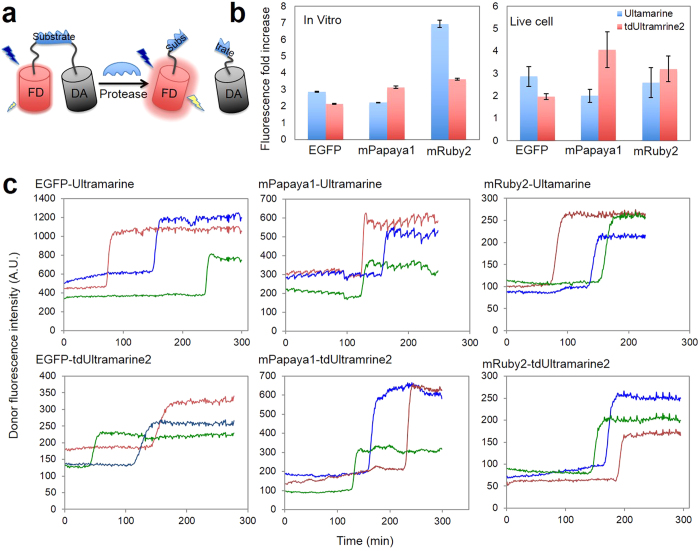
tdUltramarine2 as a dark FRET acceptor for live-cell fluorescence imaging of apoptosis. (**a**) Schematic illustration of dark acceptor-based protease biosensor based on FP-tdUltramarine2 FRET pairs. (**b**) Fluorescence intensity increase comparison of FP-tdUltramarine2 with FP-Ultramarine after protease cleavage. Fluorescence intensity fold increase defined as the fluorescence of donor at the end of protease cleavage/fluorescence of donor without protease cleavage. The end of protease cleavage was identified by SDS-PAGE. Error bars indicate the mean  ±  SEM. *In vitro* experiment were performed at least three times in triplicate. Live cells experiments average the signal change from 20–40 different cells. (**c**) Caspase-3 activation assayed by dark acceptor-based FRET described in this work. Transfected HeLa cells were treated with staurosporine (2 μM in HHBSS) and donor fluorescence was monitored over time. Representative traces for individual cells are depicted. See Supplementary Movies 1,2–3.

To quantitatively determine the FRET efficiency, purified proteins were treated with trypsin to effect cleavage of the linker and complete loss of FRET. Although trypsin cuts indiscriminately at exposed lysines and arginines, it has been used extensively in similar experiments^33^,^34^,^35^,^36^. Absorption spectra of FRET constructs (Supplementary Fig. 10a and Supplementary Fig. 11) showed that the proteins do in fact stay intact during cleavage. These experiments revealed that, to our surprise, tdUltramarine2 did not provide consistently improved performance relative to Ultramarine (Fig. 5 and Supplementary Table 3). Specifically, tdUltramarine2 provided more quenching of mPapaya, but less quenching of EGFP, relative to Ultramarine. For mRuby2, Ultramarine provided dramatically better quenching than tdUltramarine2, though the validity of this result is suspect due to the unexpectedly high acceptor to donor stoichiometry in this protein. One possible explanation for this anomalous behavior is that mRuby2 and Ultramarine (which share 65% amino acid identity) are able to form a weak heterodimer that brings the chromophores into close proximity. An analogous mRuby2-tdUltramarine2 heterodimer does not form since the fused second copy of the protein occupies the dimer interface of tdUltramarine2. One hint that protein-protein interactions are playing a role in the mRuby2-Ultramarine FRET pair is that small changes in the absorption spectrum were observed during proteolysis, but no changes were observed for mRuby2-tdUltramarine2 (Supplementary Fig. 10a).

To test the utility of tdUltramarine2 to serve as a dark FRET acceptor in mammalian cells, expression plasmids encoding six similar FRET constructs, each with the caspase-3 substrate sequence (DEVD) in the linker region, were constructed. HeLa cells transfected with each of these plasmids were treated with staurosporine to induce caspase-3 activation and fluorescence was imaged through time. As expected, the stochastic activation of caspase-3 activity resulted in abrupt increases in donor fluorescence due to the loss of FRET (Fig. 5). Generally speaking, the observed increases in fluorescence were very similar to the results obtained with purified proteins. The one exception was the mRuby2-Ultramarine FRET pair, which exhibited a 6.9-fold increase in fluorescence as purified protein, but only a 2.6-fold increase in cells. We suggest that the crowded intracellular environment may decrease the extent of heterodimerization between mRuby2 and Ultramarine and thereby lowered the initial FRET efficiency. Overall, these experiments demonstrate that tdUltramarine2 is an effective dark FRET acceptor, though it does not provide any substantial improvements relative to the original Ultramarine construct.

### Photoacoustic sensing of FRET biosensors with optimized chromoprotein acceptors

Fluorescence-based FRET biosensors have proven to be useful in a range of biotechnology applications but light-scattering in tissues and tissue autofluorescence limit their *in vivo* applications. PA imaging offers an alternative and promising means for imaging of FRET-based biosensors. Specifically, changes in FRET efficiency are expected to change the efficiency of photothermal conversion, particularly when the donor has a high quantum yield (i.e., a poor PA chromophore) and the acceptor has a low quantum yield (i.e., a good PA chromophore). Wang *et al.*^24^,^25^ have demonstrated that PA microscopy can be used for FRET imaging with enhanced penetration depth and improved spatial resolution compared to planar fluorescence imaging. However, these experiments were conducted by using high concentrations of donor- and acceptor-dyes to produce proximity-quenching effects and no FRET-biosensor constructs were evaluated.

**Figure 6:**
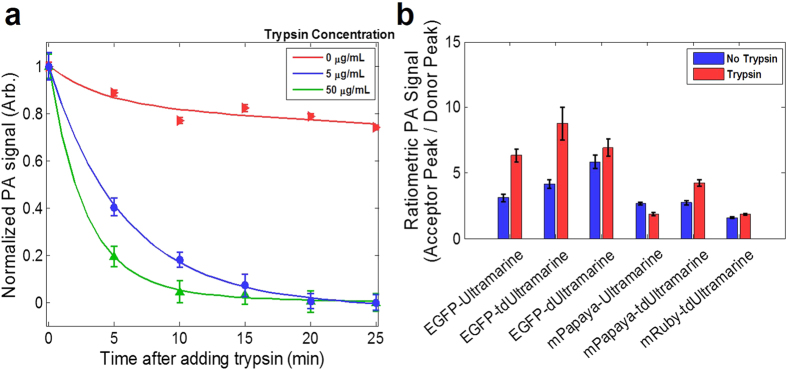
PA-based FRET imaging. (**a**) PA imaging of a FRET construct (EGFP-Ultramarine). (**b**) Ratiometric PA imaging of FRET constructs. The mPapaya-Ultramarine sample was repeated only twice, while all other samples were repeated at least three times. Error bars represent the SEM.

To evaluate whether the genetically evolved dark acceptor FRET biosensor constructs could provide photoacoustically observable changes associated with protease activity, we performed a set of multiwavelength PA imaging experiments. We first incubated the EGFP-Ultramarine construct with varying concentrations of trypsin (0, 5, and 50 μg/ml) and imaged over time at 488 nm (the donor peak) using our PA imaging setup. As shown in Fig. 6a, we observed an exponential decay in the PA signal, attributed to trypsin cleaving the FRET molecule and separating the donor (EGFP) from the acceptor (Ultramarine) thus decreasing the FRET efficiency. The control sample without trypsin has a much slower decay in PA signal that is attributed to photobleaching. Additional FRET constructs, with Ultramarine, dUltramarine2, or tdUltramarine2 as the acceptor, were purified and incubated with either PBS or trypsin for 30 minutes. The samples were imaged at both the donor and acceptor absorption peaks and the ratio was taken (signal at the acceptor peak to the donor peak, Fig. 6b). There was a general increase in the signal after trypsin was added. Direct excitation of the acceptor^24^,^25^ did occur to some degree and, for reasons that remain unclear, we observed that the PA signal at the acceptor wavelength increased upon protease cleavage. The enhanced dUltramarine2 variant gave much higher ratiometric signal increase when paired with EGFP compared with the original Ultramarine. Using the data for the extinction coefficient of blood from the literature, we estimate that a ratio of signal from oxygenated blood at 587 and 488 nm would result in a ratio of only 1.2, while our data demonstrated a ratio of up to 9-fold between the same wavelengths before cleavage improving the potential for spectrally separating these components. Upon proteolysis, the ratio increases by a maximum of 2-fold allowing for the differentiation of the protein construct before and after proteolysis.

## Discussion

While the original Ultramarine and cjBlue CPs are promising candidates for PA imaging due to low quantum yield, low absorption bleaching, high extinction coefficient, and high PA signal, we speculated that further improvement could yet be realized through the use of protein engineering. Accordingly, we optimized these two CPs for increased PA signal using a combination of visual screening and PA-based screening of large libraries of randomly generated variants. The resulting cjBlue2 and tdUltramarine2 CPs were found to have 2–4 fold improvements in PA signal. We demonstrated the utility of cjBlue2 and tdUltramarine2 for direct PA imaging and PA detection of changes in FRET efficiency. In addition, the optimized dark CP tdUltramarine2 was also utilized as a FRET acceptor for fluorescence-based sensing of protease activity, but we found tdUltramarine2 to not be substantially improved relative to the original Ultramarine protein. In contrast, dual-wavelength PA imaging of the optimized tdUltramarine2 FRET constructs resulted in significantly higher ratiometric signals than for analogous constructs incorporating Ultramarine. Further studies should be conducted to show the value of these FRET constructs in a biological setting.

One of the most important outcomes of this work is the introduction of a directed evolution methodology that promises to yield yet further improved probes for PA imaging. The directed evolution approach presented here opens up many new possibilities for PA imaging including protein engineering of optimized genetically encoded chromophores and the potential for many new reporter genes to be developed. For example, the PA-based library screening technique described here could be particularly effective for engineering new colors of CPs (optimized for PA imaging), starting from the template of a FP with a high quantum yield.

This work focuses on developing chromoproteins with absorption peaks in the shorter wavelength range between 585–610 nm, which has been applied specifically for photoacoustic microscopy applications with ~1–3mm penetration in tissue. For deeper PA imaging, such as PAT, near-infrared absorption peaks where tissue is most transparent would be more preferred. For PAT imaging, the FPs eqFP670 and E2-Crimson^37^,^38^,^39^,^40^ with their far red-shifted absorption peaks would be particularly useful and the technique described in this work could be used to enhance the photoacoustic characteristics of these FPs. Furthermore, this technique could potentially be extended to evolve further red-shifted CPs by performing multispectral PA imaging. An important future direction for this endeavor will be to use directed evolution to red-shift the absorption peaks into the near-infrared “window” where tissue is most transparent. Yet another future direction would be to extend such optimization efforts to mammalian cell-based screens. That said, reporters optimized for use in bacteria could be particularly useful for non-invasive imaging of bacterial infection with high-resolution in live animals, and for longitudinal studies of the effectiveness of antibacterial drugs to treat infections.

The fact that tdUltramarine2 does provide substantially better performance than Ultramarine for PA imaging, but not as a FRET acceptor, provides strong support for the underlying rationale of this work. Our hypothesis was that, to create a FP optimized for PA imaging, it would be critical that we screen libraries using PA imaging. Alternative approaches to screening large libraries of CPs for improved variants could potentially rely on the identification of colonies that exhibit strong visible color. Yet another approach could be to screen libraries in the context of FRET pairs and pick those colonies that exhibit the highest donor quenching. Our results suggest that such approaches would not necessarily lead to the identification of CPs that are optimal for PA imaging.

The enhanced signal of dUltramarine2 could be attributable to a number of factors including an increased extinction coefficient, lower quantum yield, increased efficiency of protein folding and chromophore maturation, decreased cytotoxic effects on the *E. coli* cells, or higher expression levels in the colonies. Although, all these effects indicate better potential for PA imaging, it is important to note that the improvement may not come entirely from a higher extinction coefficient and lower quantum yield. Notably, factors other than the quantum yield and extinction coefficient seem to be acting in concert to provide the increased SNR. Additional work is warranted to better understand this phenomenon. One hypothesized mechanism is the potential influence of lifetime on PA signals. As PA initial pressures will depend on the rate of heating it may be expected that the excited state to ground-state non-radiative decay lifetime will in part influence heating rate.

In conclusion, we present a novel method to screen and evolve genetically-encoded proteins with enhanced PA characteristics. We expect that the screening technique described here will open many avenues for the development of PA reporter molecules that will prove useful for deep-tissue, non-invasive, *in vivo* PA imaging and FRET-based biosensing.

## Methods

### General methods

All synthetic DNA oligonucleotides used for cloning and library were purchased from Integrated DNA Technologies (Coralville, IA). Miniprep plasmid DNA, polymerase chain reactions (PCR), restriction enzyme digestion, ligation and agarose gel electrophoresis were performed according to Sambrook *et al.*^41^ *Pfu* DNA polymerase was obtained from Fermentas used for regular PCR and the QuikChange Multi kit was purchased from Agilent Technologies used for site-directed mutagenesis. All restriction enzymes were obtained from Fermentas or New England Biolabs. T4 DNA ligase was obtained from Life Technologies. PCR and digestion products were purified with the QIA quick gel extraction kit (QIAGEN, Valencia, CA) or GeneJET gel extraction kit (Fermentas) according to the manufacturer’s instructions. All sequencing was performed at the Molecular Biology Service Unit at the University of Alberta or University Core DNA Services at the University of Calgary. All filters for fluorescence imaging were purchased from Chroma Technology (Rockingham, VT), Omega Filters (Brattleboro, VT), or Semrock (Rochester, NY).

### Purified protein and *E. coli* sample preparation

*E. coli* cells were transformed by electroporation with the vectors containing either mCherry (pBAD/His B vector from Life Technologies), EYFP (enhanced yellow FP) (pRSET-B vector from Life Technologies), REACh (pRSET-B), Ultramarine (pQE9N vector), or cjBlue (pRSET-B). A 4 ml culture, inoculated with a single *E. coli* colony, was grown overnight before being diluted into 250 ml of media (LB medium for mCherry, EYFP, and REACh; M9 medium for cjBlue; Terrific broth medium for Ultramarine) containing 0.1 mg/ml ampicillin. This culture was grown in 250 ml baffled shake flasks (37 °C and 225 rev./min) to the desired optical density (0.6–0.8). Protein expression was induced by adding either 0.02% L-arabinose (mCherry), 1 mM IPTG (EYFP and REACh), or 0.2 mM isopropyl 1-thio-β-D-galactopyranoside (Ultramarine and cjBlue). The *E. coli* cells were then cultured at 28 °C for 24 hours for EYFP and REACh, 30 °C for 24 hours for mCherry and Ultramarine, or 30 °C for 48 hours for cjBlue. The *E. coli* was either pelleted by centrifugation (10,000 g for 5 min) for PA imaging experiments or plated on an agar dish for PA screening (described below).

Purified proteins were used to compare optical and PA characteristics of the protein. For purification of the mCherry, EYFP, and REACh proteins, the cultured bacteria were subjected to centrifugation (10,000 g for 5 min), then resuspended in 30 mM Tris-HCl buffer (pH 7.4). French press was used to lyse the bacteria and the bacterial lysate was subjected to centrifugation (13,000 g for 45 mins at 4 °C). After centrifugation, the supernatant was collected and Ni-NTA affinity chromatography (Agarose Bead Technologies) was then applied to extract purified proteins. Purified proteins from Ni-NTA affinity chromatography were then subjected to dialysis into 10 mM MOPS, 100 mM KCl, pH 7.2 for measurement.

For purification of the Ultramarine and cjBlue CPs, the pelleted *E. coli* cells were lysed by a cell disruptor (Constant Systems), purified by Ni-NTA chromatography (Amersham), then dialyzed into phosphate buffer saline (PBS, pH 7.4).

### Random mutagenesis and *E. coli* plate preparation

Random mutagenesis was performed by error-prone polymerase chain reaction (PCR) as described in Fromant *et al.*^42^. In brief, Ultramarine in pQE9N (Qiagen) and cjBlue in pRSET-B (Life Technologies) were amplified with a 5′ XhoI forward primer and a 3′ HindIII reverse primer using *Taq* polymerase (New England Biolabs) in the presence of MnCl_2_. Full-length gene libraries were digested with XhoI/HindIII (Fermentas) and ligated into similarly digested pBAD/His B plasmid with T4 DNA ligase (Life Technologies). Plasmid libraries were expressed in *E. coli* strain DH10B (Life Technologies) on LB (Luria-Bertani) agar plates supplemented with 0.1 mg/ml ampicillin and 0.02% L-arabinose at 37 °C overnight. Fresh 0.2% agar was overlaid on the top of colonies prior to PA screening to mitigate contamination.

### Absorbance-based screening

*E. coli* colonies expressing the Ultramarine or cjBlue libraries were grown on 10 cm Petri dishes. The top 20–30 colonies with darkest color were manually picked into 4 ml LB media supplemented with 0.1 mg/ml ampicillin and 0.02% L-arabinose and incubated overnight. Crude protein extracts were obtained by taking advantage of B-PER Protein Extraction Reagen’t (Pierce/Thermo Scientific). 100 μL crude protein extracts were subject to absorption test and spectra (400–800 nm wavelength scan) were recorded by a DU-800 UV-visible spectrophotometer (Beckman). After absorption maximum comparison, 5–10 winners were picked and used as a template for the next round of mutagenesis.

### Construction of tandem Ultramarine 7.2 dimer

To construct a tandem dimer, dimer Ultramarine 7.2 in pBAD/His B was amplified in two separate PCR reactions. In the first reaction, 5′ XhoI and 3′ PstI restriction sites were introduced. In the second reaction, 5′ PstI with a linker and 3′ HindIII were introduced. Three-way ligation strategy provided a tandem gene of the form A-linker-A in the XhoI/HindIII sites of pBAD/His B, where the linker was a 13-residue sequence (SCSGTGSTGSGSS) that included a PstI restriction site.

### Protein characterization

Absorption spectra of the original and variant protein structures were measured by placing dilute samples into 1 cm quartz microcell cuvettes into a DU-800 UV-visible spectrophotometer (Beckman). Molar extinction coefficients were determined by using the absorption measurements for serial dilutions of the protein, the known concentration of proteins, and the Beer-Lambert equation.

mCherry was used as a reference to determine the quantum yield of CPs. Briefly, serial dilutions of the reference and proteins were created with absorbance ranging from 0.01–0.1. The fluorescence spectra of the samples were recorded using a QuantMaster spectrofluorometer (Photon Technology International). The quantum yield could be determined by using the equation Φ_sample_ = Φ_reference_ × (S_sample_/S_standard_), where Φ represents the quantum yield and S represents the slope of curves obtained by plotting the total fluorescence intensity (integrated over wavelength) versus the absorbance.

### Photoacoustic system

Figure 1b shows a schematic of the experimental setup. A 10 Hz, Q-switched Nd:YAG laser (SLIII-10, Continuum) and an optical parametric oscillator (SL OPO Plus, Continuum) were used to generate wavelengths extending from 450 to 700 nm. The light was coupled into a light guide (CeramOptec Industries, 900 fibres [185 μm core diameter, 200 μm cladding diameter, 250 μm jacket diameter], NA = 0.26/0.37 ± 0.02) with one input and ten outputs to direct the light to the sample, homogenize the beam shape, and illuminate the sample uniformly. The outputs of the light guide were arranged concentrically around a 25 MHz, 12.7 mm focused ultrasound transducer (V324-SM, Olympus Panametrics-NDT) such that the center of the illumination spot was aligned with the focus of the transducer. The holder for the transducer and light guide was mounted on a motorized stage for raster scanning and positioning of the transducer. A camera was used to visualize the samples, verify the alignment of the ultrasound and sample, and detect the *E. coli* colonies on the agar plates. To automatically determine the location of the colonies a circle-based Hough transform was used. This allowed point-to-point (colony-to-colony) scanning rather than raster scanning which reduce the time required per plate.

The acoustic and PA signals detected by the transducer were amplified by 39 dB using a pulser-receiver unit (5073PR, Olympus Panametric NDT) and recorded with a data acquisition card (CS8229, Gage Applied Technologies). For interlaced PA and ultrasound imaging, a digital input-output card (NI CB-2162, National Instruments) was used to synchronize the ultrasound and laser systems, similar to previous work^43^. A photodiode signal was recorded and used to normalize the PA signals.

To determine the PA characteristics, purified proteins or resuspended *E. coli* cells (1–10 × 10^9^cells/ml) were diluted in PBS and injected into a 1.57 mm inner diameter tube (PE-205, Intramedic). The tubes were sealed and positioned beneath the transducer for M-mode scans (one-dimensional imaging over time), B-scan (two-dimensional imaging), and C-scan (three-dimensional imaging). To screen the *E. coli* plates, custom software was designed to integrate the data acquisition card, digital input-output card, motion controller, and camera to automatically detect and position the transducer above the *E. coli* colonies.

Animal imaging was performed by injecting 10 μL of 10^7^–10^8^cells/ml *E. coli* cells producing CPs or their variants into the hindlimb of a rat sacrificed just prior to the imaging or by injecting 10 μL of 10^7^–10^8^ cells/ml CP-producing *E. coli* cells into the ear of live rats. The rat was anaesthetized using isofluorane. PA imaging started within 30 minutes of the injection. All animal experiments were conducted in accordance to the protocols set out by the Animal Care and Use Committee at the University of Alberta.

### Signal and image processing

The detected PA signals were first processed using a Hilbert-transform envelope detection algorithm. SNR was calculated using the average maximum value of the enveloped PA signal divided by the noise standard deviation. For image display of C-scans, the maximum values of each enveloped signal was used and interpolated using linear interpolation. To separate different optically-absorbing molecules such as blood and Ultramarine or cjBlue, a demixing algorithm was implemented using a constrained, non-negative, least-squares regression algorithm. All post-processing was done in MATLAB.

### Construction of protease biosensor for *in vitro* test

To construct tdUltramarine2 fusions to EGFP, mPapaya1 and mRuby2 for testing protease activity, the fluorescent donor genes were amplified with a 5′ XhoI forward primer and a 3′ KpnI containing a linker 1 reverse primer in three separate PCR reactions. This linker 1 was a 10-residue peptide GSGDEVDGGT, where DEVD is the substrate sequence for caspase-3. The C-terminus of the N-terminal FP ends in a lysine residue, which served as the substrate recognition site for trypsin. Meanwhile, dUltramarine2 in pBAD/His B was amplified in two separate PCR reactions. In the first reaction, 5′ KpnI and 3′ PstI restriction sites were introduced. In the second reaction, 5′ PstI with a linker 2 and 3′ HindIII were introduced, where the linker 2 was a 13-residue SCSGTGSTGSGSS including PstI restriction site, see 2.4.5 (construction of tdUltramarine2). A four-way ligation strategy provided a form A-linker 1-B-linker 2-B in XhoI/HindIII sites of spBAD/His. spBAD/His B is equivalent to the pBAD/His B plasmid with mutation A2056C in pBR322 origin. This unexpected mutation was found to increase the plasmid replication at least 2-fold.

To construct FP (*i.e.*, EGFP, mPapaya1 and mRuby2) plus Ultramarine FRET constructs, the gene encoding Ultramarine was amplified with a 5′ KpnI forward primer and 3′ HindIII reverse primer. A three-way ligation (A-linker 1-B) was performed in XhoI/HindIII sites of spBAD/His B. To achieve complete cleavage by trypsin for FRET (mPapaya 1-CP and EGFP-CP), we substituted the DE amino acids in linker 1 with KK.

### Construction of caspase-3 biosensor for live cell imaging

For mammalian cell expression of FRET construct, a modified pcDNA3.1(+) vector developed by Dr. Yidan Ding was utilized^44^. After modification, the vector has XhoI and HindIII restriction sites in the same reading frame as the same site in pBAD/His B vector. All the FRET constructs containing caspase-3 cleavage substrate DEVD were treated with XhoI and HindIII, and ligated with similarly treated modified pcDNA3.1(+) vector.

### General methods for the live cell imaging

All the mammalian cells expression plasmids were purified using a Plasmid Miniprep kit (Qiagen). Hela cells (CCL2 line; ATCC) were maintained in Dulbecco’s Modified Eagle Medium (DMEM) (Life Technologies) supplemented with 10% fetal bovine serum (FBS) (Sigma), 2 mM GlutaMax (Life Technologies) and penicillin-streptomycin at 37 °C and 5% CO_2_ according to standard procedures. Transient transfection was carried out using Turbofect^TM^ (Fermentas) according to manufacturer’s instructions. Generally, cells in 35 mm imaging dishes were transfected with 1 μg plasmid DNA mixed with 2 μL of transfection reagent. Imaging was performed after 24–48 h post-transfection at room temperature in HEPES-buffered Hank’s Balance Salt Solution (HHBSS).

### Imaging of staurospaurine-induced apoptosis

To initiate apoptosis, cells were treated with 2 μM staurosporine and incubated another 60–90 min before imaging. Imaging was conducted on an Axiovert 200 M microscopy (Zeiss) equipped with a 75 W xenon-arc lamp and 20× objective lens (NA = 0.75, air) and a 14-bit CoolSnap HQ2 cooled CCD camera (Photometrics), driven by open source Micro-Manager software. For a typical experiment, images were recorded at 1 minute intervals.

## Additional Information

**How to cite this article**: Li, Y. *et al.* Engineering Dark Chromoprotein Reporters for Photoacoustic Microscopy and FRET Imaging. *Sci. Rep.* **6**, 22129; doi: 10.1038/srep22129 (2016).

## Supplementary Material

Supplementary Information

Supplementary Movie 1

Supplementary Movie 2

Supplementary Movie 3
