# Supplementary material for: Engineering Dark Chromoprotein Reporters for Photoacoustic Microscopy and FRET Imaging
Source: Sci Rep. 2016 Mar 1;6:22129. doi: 10.1038/srep22129 (PMC4772073; doi:10.1038/srep22129)
Supplement: Supplementary Information [file srep22129-s1.pdf]

Engineering Dark Chromoprotein Reporters for Photoacoustic Microscopy and FRET Imaging

Yan Li[1,*], Alex Forbrich[2,*], Jiahui Wu[1], Peng Shao[2], Robert E. Campbell[1] & Roger Zemp[2]

[1] Department of Chemistry, University of Alberta, Edmonton, Alberta, Canada T6G 2G2.

[2] Department of Electrical & Computer Engineering, University of Alberta, Edmonton, Alberta, Canada T6G 2V4. *These authors contributed equally to this work.

Correspondence and requests for materials should be addressed to R.E.C. (email: robert.e.campbell@ualberta.ca) or to R.Z. (email: rzemp@ualberta.ca).

Supplementary Figures

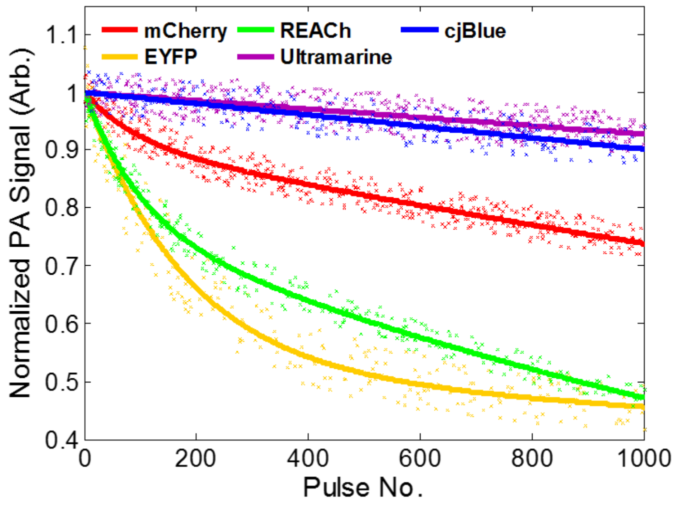

Supplementary Figure 1. Photostability of FPs and CPs. The normalized PA signal from a series of 2.5 mJ/cm^2 laser pulses for purified mCherry (red, $\lambda_{exc.}$ = 585 nm), EYFP (yellow, $\lambda_{exc.}$ = 514 nm), REACh (green, $\lambda_{exc.}$ = 514 nm), Ultramarine (purple, $\lambda_{exc.}$ = 585 nm), and cjBlue (blue, $\lambda_{exc.}$ = 585 nm) proteins is shown.

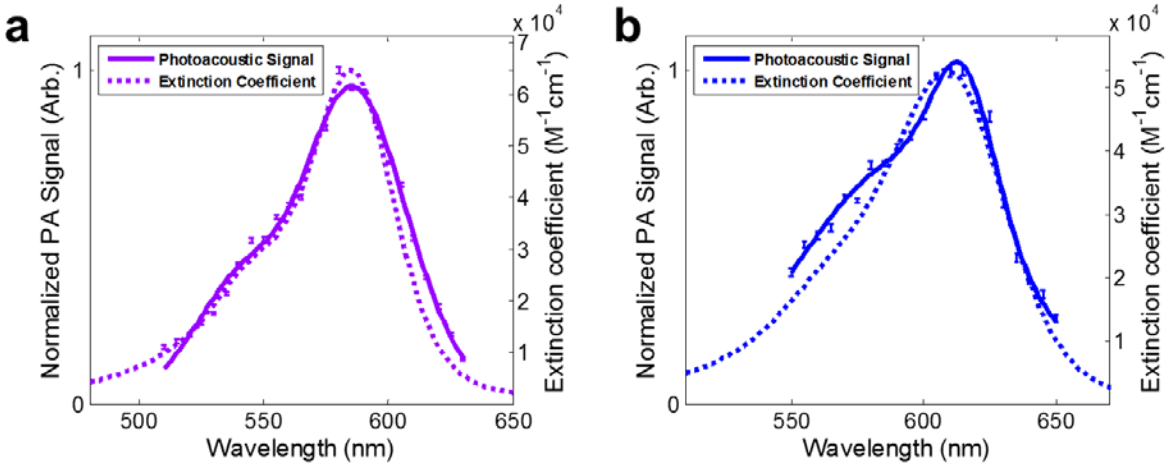

Supplementary Figure 2. Spectra of CPs. PA (solid) and absorption (dashed) spectra of (**a**) Ultramarine and (**b**) cjBlue. The mean is determined from three trials with the each trial using at least 20 laser pulses at each wavelength. Error bars represent the standard error of the mean (SEM).

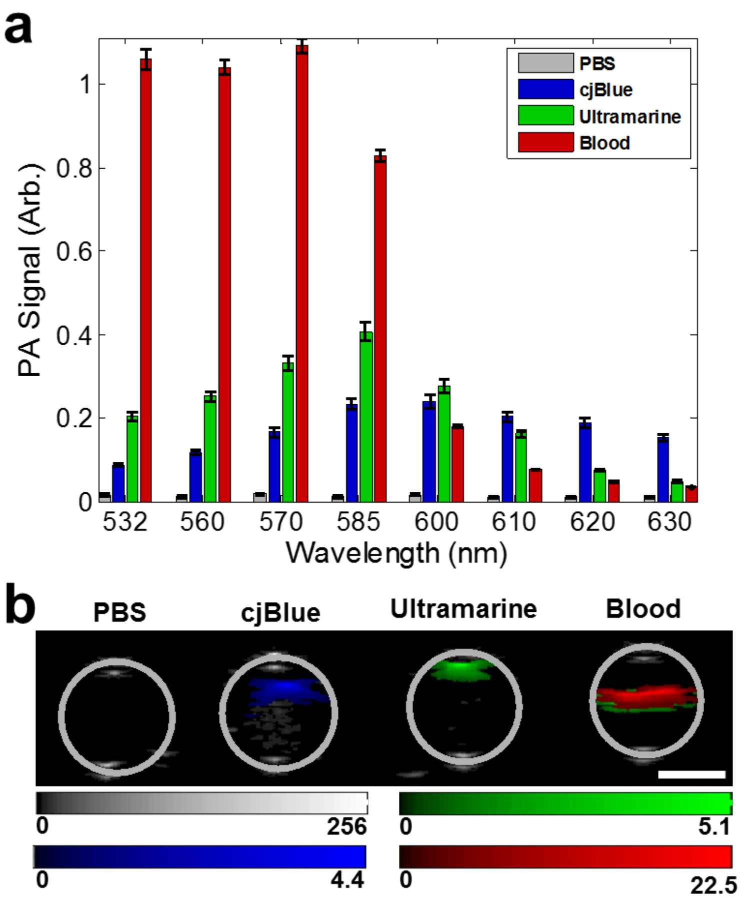

Supplementary Figure 3. Multi-wavelength B-scan studies of tubes containing PBS, cjBlue or Ultramarine *E. coli* cells (~10^9 cells/mL), or heparinized rat blood. (**a**) Average maximum PA signal within each tube. The mean is determined from at least 20 laser pulses at each wavelength. Error bars represent the SEM. (**b**) Spectrally unmixed B-scan image of the tubes (from left to right – PBS, cjBlue, Ultramarine, blood). The grayscale colormap represents the ultrasound intensity, while the blue, green, and red colormaps represent the relative concentration of cjBlue, Ultramarine, oxygenated hemoglobin, respectively. Scale bar represents 1 mm.

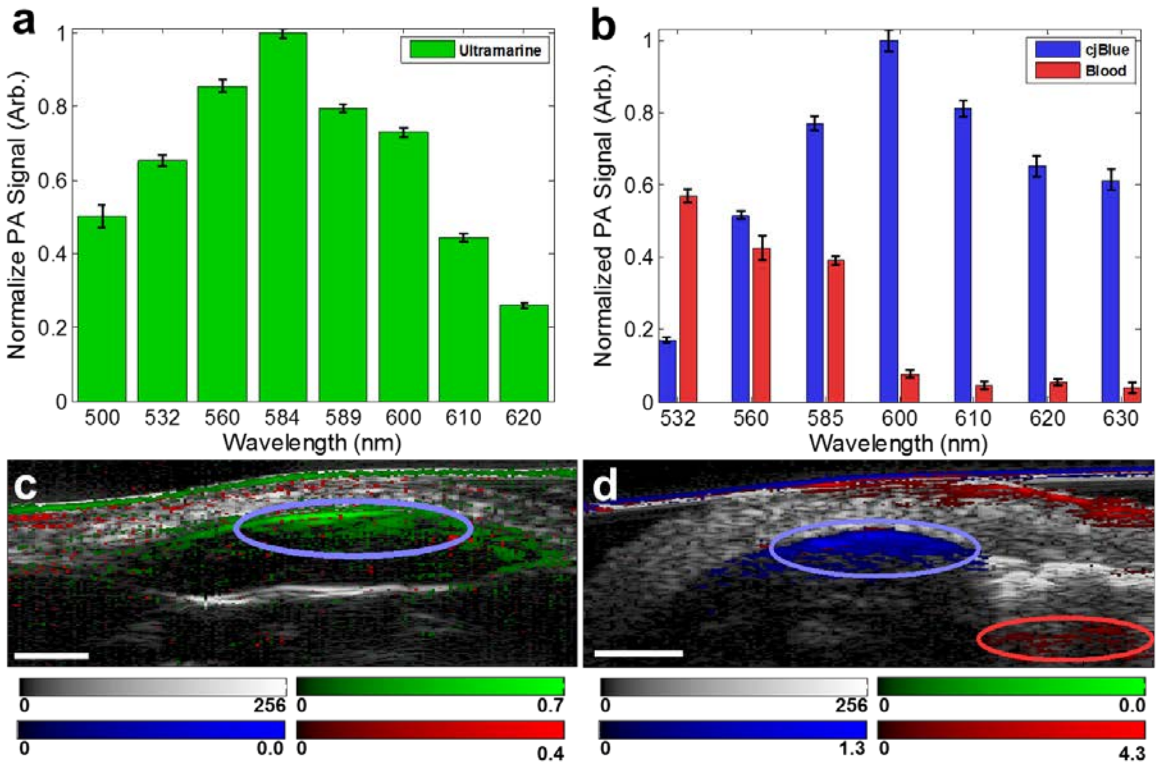

Supplementary Figure 4. *In situ* multispectral PA imaging and spectral unmixing. *E. coli* cells (~10^9 cells/mL) producing either Ultramarine (**a,c**) or cjBlue (**b,d**) were injected into a recently sacrificed rat. (**a**) Normalized average PA signal from the Ultramarine injection site of animal 1. (**b**) Normalized average PA signal from the cjBlue injection site and a region of interest thought to be blood in animal 2. For (**a**) and (**b**) the mean is determined from at least 20 laser pulses at each wavelength. Error bars represent the SEM. (**c**) Spectrally unmixed B-scan image of animal 1 with the site of Ultramarine bacteria injection indicated by a blue ellipse. (**d**) Spectrally unmixed B-scan image of animal 2 with the cjBlue bacteria injection site indicated by a blue ellipse and a region that corresponds to blood indicated with a red ellipse. The gray colormap represents the ultrasound intensity, while the blue, green, and red colormaps represent the relative concentration of cjBlue, Ultramarine, oxygenated hemoglobin, respectively. Scalebar represents 1mm.

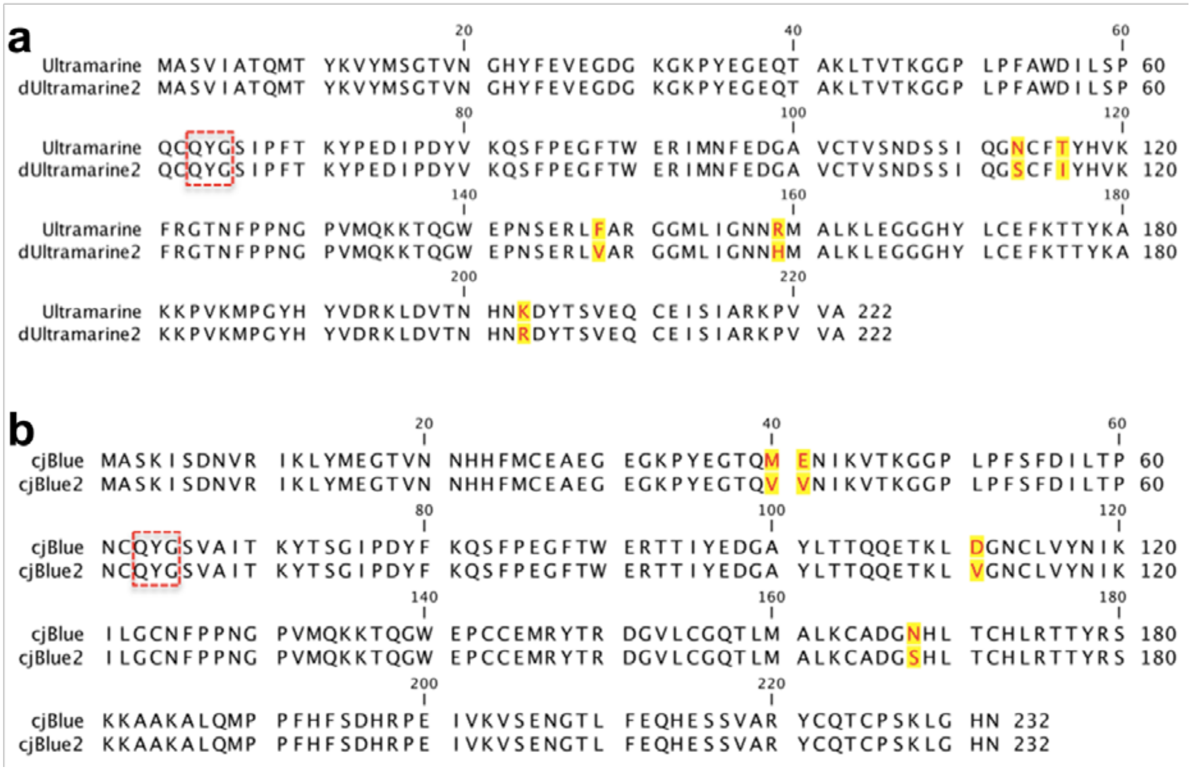

Supplementary Figure 5. (**a**) Sequence alignment of Ultramarine and dUltramarine2. (**b**) Sequence alignment of cjBlue and cjBlue2. Substitutions are represented as red text on a yellow background. The chromophore forming residues are boxed with a dashed red line.

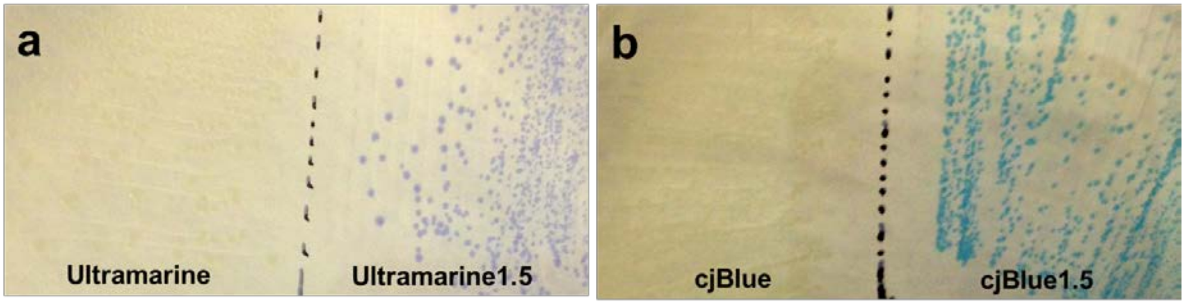

Supplementary Figure 6. (**a**) Comparison of *E. coli* expressing Ultramarine (left) with dUltramarine1.5 (right). (**b**) Comparison of *E. coli* expressing cjBlue (left) with cjBlue1.5 (right). Photographs of the agar plates were taken 12 hours after the bacteria had been transformed with the expression plasmid.

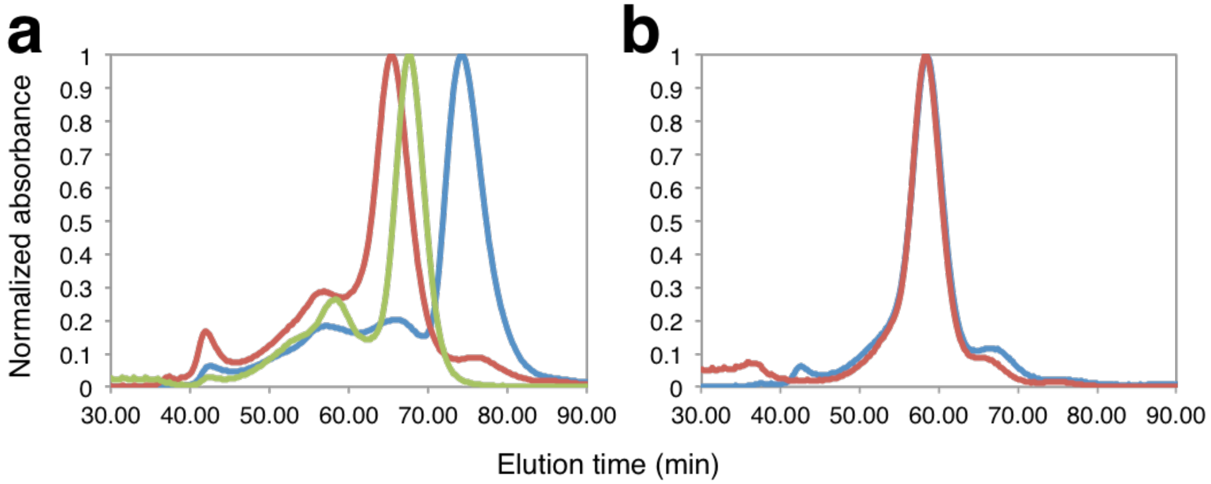

Supplementary Figure 7. Characterization of the oligomeric structure of CPs. (**a**) Ultramarine (blue), dUltramarine2 (red) and tdUltramarine2 (light green) size-exclusion chromatography elution profiles. (**b**) cjBlue (blue) and cjBlue2 (red) size-exclusion chromatography elution profiles. Proteins purified by Ni-NTA chromatography (500µl with concentration ~30µM) were subjected to gel filtration chromatography on a HiLoad 16/60 Superdex 75 pg gel filtration column with absorbance-based detection at 280 nm.

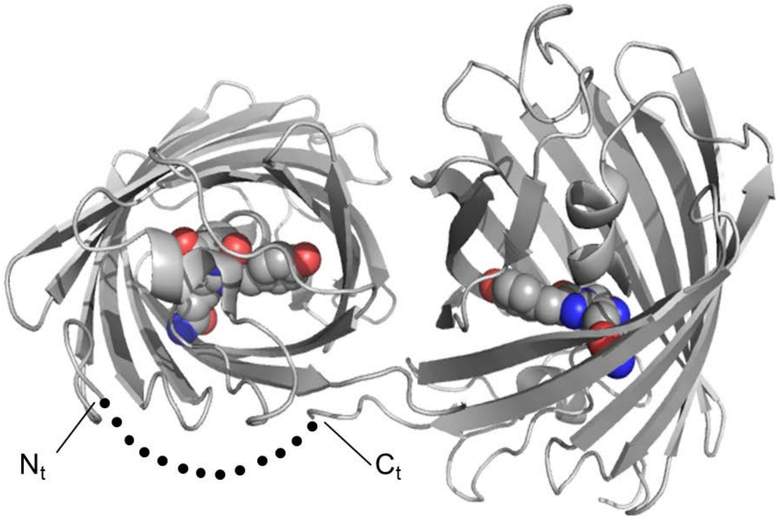

Supplementary Figure 8. Graphical representation of tdUltramarine2. The X-ray crystal structure of Rtms5-H146S (PDB ID 2P4M) in high pH[1] is used here to represent dUltramarine2. The intersubunit linker (SCSGTGSTGSGSS) between the N-terminus (N_t) and C-terminus (C_t) is represented as a dotted line.

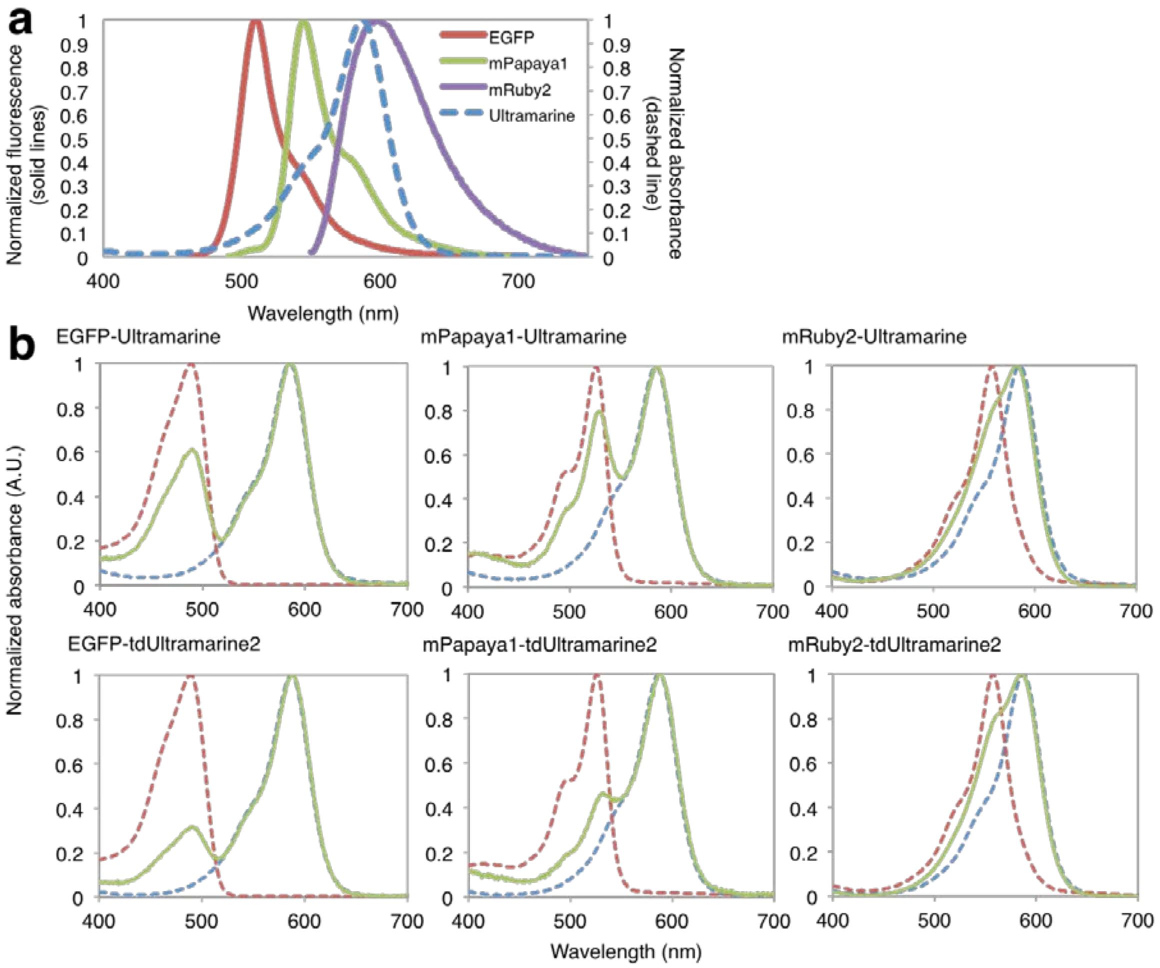

Supplementary Figure 9. (**a**) Spectral overlap of the absorbance spectrum of tdUltramarine2 (dashed blue line) with the fluorescence emission spectra of EGFP (red), mPapaya1 (green) and mRuby2 (purple). (**b**) Absorption spectrum of FRET donor-acceptor fusion constructs (solid green line) containing either Ultramarine or tdUltramarine2, overlaid with the absorption spectra of the donor alone (dashed red line) and acceptor alone (dashed blue line).

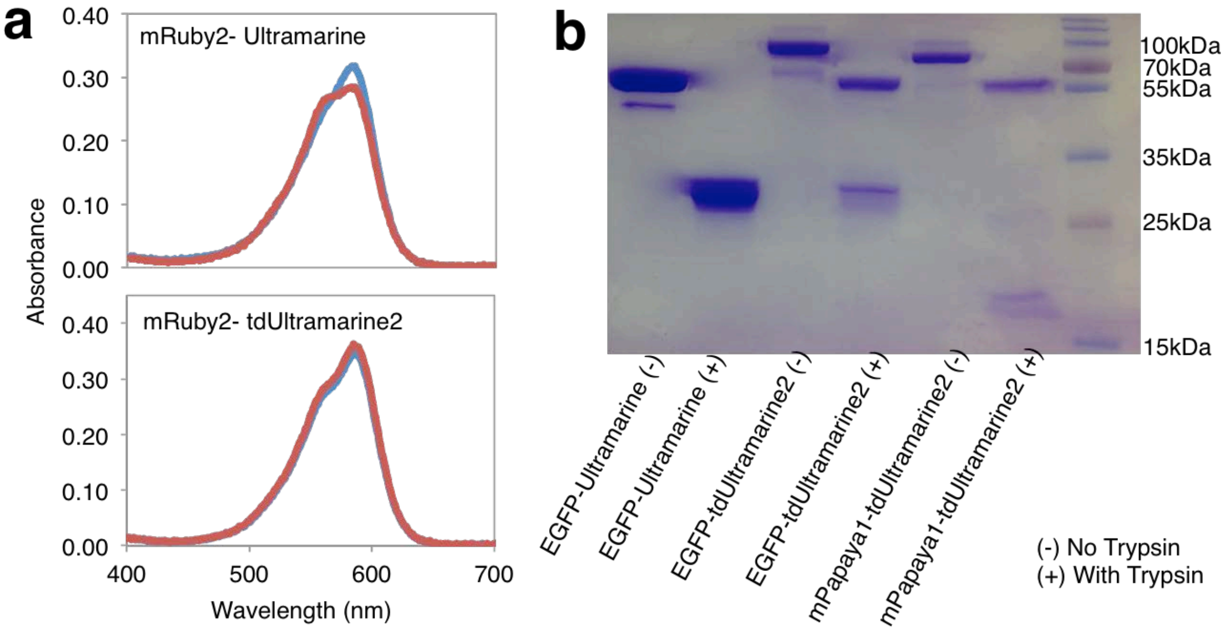

Supplementary Figure 10. (**a**) Absorption spectra of mRuby2-Ultramarine (top panel) and mRuby2-tdUltramarine2 (bottom panel) before (blue line) and after (red line) protease cleavage. (**b**) SDS-PAGE analysis of FRET fusion constructs before and after trypsin lysis. EGFP-Ultramarine (53 kDa), EGFP-tdUltramarine2 (79 kDa), mPapaya1-tdUltramarine2 (79 kDa), EGFP (27 kDa), mpapaya1 (27 kDa), Ultramarine (25 kDa), tdUltramarine2 (58 kDa).

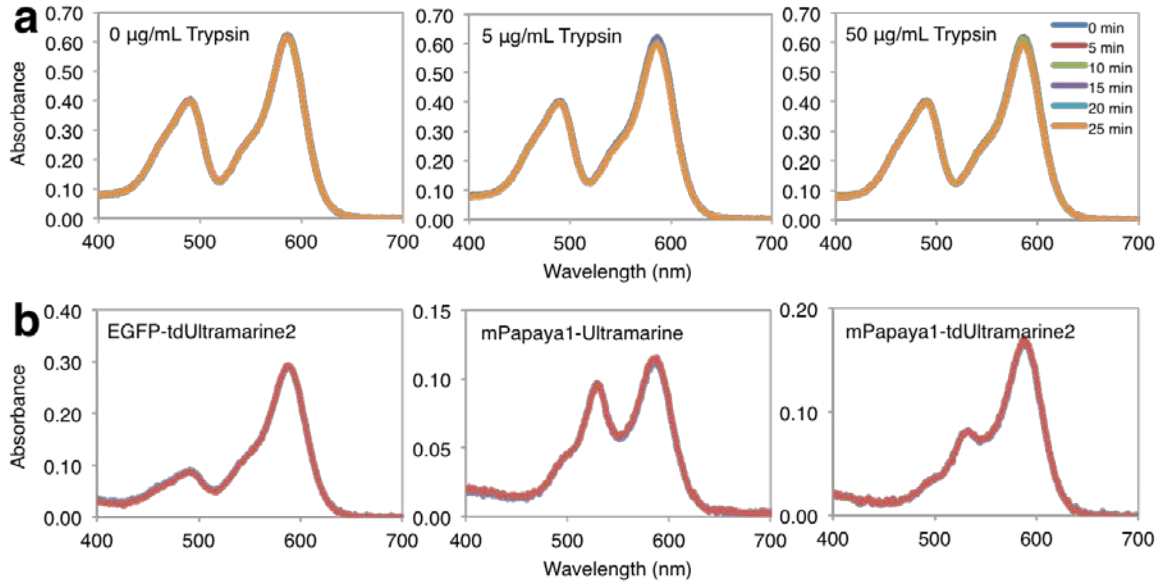

Supplementary Figure 11. (**a**) Monitoring the absorption spectra of EGFP-Ultramarine for 25 minutes with 0, 5 and 50 ug/ml of Trypsin cleavage. (**b**) Absorption spectra of EGFP-Ultramarine, EGFP-tdUltramarine2, mPapaya1-Ultramarine, mPapaya1-tdUltramarine2 before (blue line) and after (red line) Trypsin cleavage.

Supplementary Tables

Supplementary Table 1. Spectral characteristics of selected FPs and CPs

Protein	$\lambda_{exc.}$ (nm)	ε_{max} * (10^3 $M^{-1}cm^{-1}$)	QY	SNR ** (dB)	SNR/ε_{Max} ** 10^{-6} (dB) / ($M^{-1}cm^{-1}$)	Reference
mCherry	587	72.0	0.22	10.1	140	[2,3]
EYFP	514	83.4	0.61	-1.9	23.2	[4]
REACh	513	100.1	0.02	18.6	186	[5]
cjBlue	610	52.7 (66.7) ǂ	< 0.0001	36.1	541	[6]
Ultramarine	587	64.4 (64.0) ǂ	0.001	46.4	724	[7]

* ε_{max} is based on the monomer concentration

** SNR is for 100 μM of protein using 2.5 mJ/cm^2 laser fluence at the peak absorption wavelength.

ǂ Number provided is our measurement, followed by the literature value in parentheses.

Supplementary Table 2. Optical characteristics of evolved CPs

Protein	$\lambda_{exc.}$ (nm)	ε_{max}* (10^3 $M^{-1}cm^{-1}$)	QY	Oligomeric state	SNR** (dB)
Ultramarine	585	64.4ǂ	0.001†	monomer	46.4
dUltramarine2	587	81.5	< 0.0001	dimer	50.4
tdUltramarine2	587	203.4	< 0.0001	tandem dimer	57.4
cjBlue	610	52.7ǂ	< 0.0001	tetramer	36.1
cjBlue2	603	56.6	< 0.0001	tetramer	42.4

* ε_{max} is based on the monomer concentration

** SNR is for 100 μM of protein using 2.5 mJ/cm^2 laser fluence at the peak absorption wavelength.

ǂ The reported extinction coefficients of Ultramarine and cjBlue are 64,000 $M^{-1}cm^{-1}$ and 66,700 $M^{-1}cm^{-1}$, respectively[6,7].

† The reported quantum yield of Ultramarine is 0.001(Ref. 7).

Supplementary Table 3. Fluorescence intensity increases of different FRET pairs after protease cleavage

FRET pair	R_0 (nm)*	FRET pair stoichiometry (#acceptor / #donor)**	*In vitro* fold increase (FRET efficiency)	Live cell fold increase (FRET efficiency; # cells)
EGFP-Ultramarine	5.1	1.6	2.9 (65%)	2.9 (65%; n = 26)
EGFP-tdUltramarine2	6.0	1.0	2.1 (53%)	2.0 (49%; n = 20)
mPapaya1-Ultramarine	6.1	1.3	2.2 (55%)	2.0 (50%; n = 22)
mPapaya1-tdUltramarine2	7.4	0.9	3.1 (68%)	4.1 (75%; n = 28)
mRuby2-Ultramarine	5.4	4.6	6.9 (86%)	2.6 (61%; n = 13)
mRuby2-tdUltramarine2	6.5	1.5	3.6 (72%)	3.2 (69%; n = 18)

* R_0 values were calculated as previously described[8].

FRET pair stoichiometry was calculated using the ε values in **Supplementary Table 4.

Supplementary Table 4. Spectral properties of fluorescent donors

**Protein**	**$\lambda_{abs.}$ (nm)**	**λ_{em} (nm)**	**ε (10^3 $M^{-1}cm^{-1}$)**	**QY**
EGFP	488	507	56	0.60
mPapaya1	530	541	43	0.81
mRuby2	559	600	113	0.38

Supplementary Movie Legends

Supplementary Movie 1. Live-cell video of staurosporine-induced apoptosis of EGFP-tdUltramarine2.

Supplementary Movie 2. Live-cell video of staurosporine-induced apoptosis of mPapaya-tdUltramarine2.

Supplementary Movie 3. Live-cell video of staurosporine-induced apoptosis of mRuby2-tdUltramarine2.

References

1. Battad, J. M. *et al.* A structural basis for the pH-dependent increase in fluorescence efficiency of chromoproteins. *J. Mol. Biol.* **368,** 998–1010 (2007).

2. Shaner, N. C. *et al.* Improved monomeric red, orange and yellow fluorescent proteins derived from Discosoma sp. red fluorescent protein. *Nat. Biotechnol.* **22,** 1567–1572 (2004).

3. Shu, X., Shaner, N. C., Yarbrough, C. A., Tsien, R. Y. & Remington, S. J. Novel chromophores and buried charges control color in mFruits. *Biochemistry* **45,** 9639–9647 (2006).

4. Siegel, R. M. *et al.* Measurement of molecular interactions in living cells by fluorescence resonance energy transfer between variants of the green fluorescent protein. *Sci. STKE* **2000,** pl1 (2000).

5. Ganesan, S., Ameer-Beg, S. M., Ng, T. T. C., Vojnovic, B. & Wouters, F. S. A dark yellow fluorescent protein (YFP)-based Resonance Energy-Accepting Chromoprotein (REACh) for Förster resonance energy transfer with GFP. *Proc. Natl. Acad. Sci. U. S. A.* **103,** 4089–4094 (2006).

6. Chan, M. C. Y. *et al.* Structural characterization of a blue chromoprotein and its yellow mutant from the sea anemone Cnidopus japonicus. *J. Biol. Chem.* **281,** 37813–37819 (2006).

7. Pettikiriarachchi, A., Gong, L., Perugini, M. A., Devenish, R. J. & Prescott, M. Ultramarine, a chromoprotein acceptor for Förster resonance energy transfer. *PLoS One* **7,** e41028 (2012).

8. Clegg, R. M. Forster resonance energy transfer — FRET what is it, why do it, and how it's done. In *Laboratory Techniques in Biochemistry and Molecular Biology* vol. **33,** (eds. Gadella, T. W. J.) 1.1-1.57 (Academic Press, Burlinton, Canada, 2009).
